# End‐of‐life decision‐making of dairy cattle and calves: A survey of British farmers and veterinary surgeons

**DOI:** 10.1002/vro2.51

**Published:** 2022-11-25

**Authors:** Joseph M. Neary, Cherry Bedford, Robert F. Smith

**Affiliations:** ^1^ Department of Livestock and One Health Institute of Infection, Veterinary and Ecological Sciences Leahurst Campus, University of Liverpool Neston UK

## Abstract

**Background:**

The study aim was to characterise issues faced by farmers and veterinary surgeons when making end‐of‐life decisions for dairy cattle.

**Methods:**

Online surveys were distributed to British dairy farmers and veterinary surgeons for 20 weeks from November 2020.

**Results:**

There were 83 responses (37 farmers, 46 veterinary surgeons). Among youngstock, the risk of unassisted/natural death (2.6% ± 0.3%) was almost double the risk of euthanasia (1.4% ± 0.3%; *p* = 0.003). The opposite, however, was true in the milking herd: the risk of euthanasia (2.3% ± 0.3%) was greater than unassisted/natural death (1.6% ± 0.2%; *p* = 0.05). A fallen stock collector (62%) typically performed euthanasia and most farms (66%) did not have anyone trained to perform euthanasia. Most deaths within the milking herd were attributed to ‘unknown or not recorded’ (median 15% of deaths). The factors that farmers most frequently reported as strongly influencing their decision of when to euthanase an animal relative to the onset of disease were ‘failure to respond to treatment’ (89%), ‘veterinary advice’ (89%) and ‘severity of disease’ (88%). On average, veterinarians had moderate or high confidence that 60% of dairy farm clients ‘are performing euthanasia in a timely manner so as to prevent unnecessary suffering’. Veterinary surgeons had variable agreement on the time to euthanasia for various conditions.

**Conclusions:**

The survey highlighted end‐of life decision‐making successes and areas for improvement on dairy farms. An evidence‐based, decision‐support framework may help end‐of‐life decision‐making, particularly for complex diseases.

## INTRODUCTION

A study of expert opinions reported that delayed euthanasia of livestock was a leading animal welfare issue.^1^ Guidance provided by British farm industry stakeholders, such as Red Tractor farm assurance (redtractor.org.uk) and the British Cattle Veterinary Association (https://www.bcva.org.uk), aim to reduce end‐of‐life animal suffering by advocating immediate euthanasia when an animal is in obvious distress. Unless ‘the animal is in such distress that immediate euthanasia is required’, the best course of action, however, may not be clear. Furthermore, there is ambiguity surrounding what constitutes ‘timely euthanasia’ and ‘animal distress’; there has been limited study of the barriers for dairy cattle euthanasia.^2^

The welfare of the animal should be at the forefront of decision‐making, but as the Farm Animal Welfare Committee reported, there are numerous factors that likely influence on‐farm euthanasia decisions. These include farmer/veterinary surgeon perception of the severity of the illness, likelihood of recovery, the level of pain and distress the animal is suffering, also farmer willingness and confidence to euthanase.^3^ The aim of this study was to characterise the factors influencing farmer and veterinary surgeon end‐of‐life decision‐making in dairy cattle and youngstock.

## MATERIALS AND METHODS

Dairy farmers in the United Kingdom, who considered themselves responsible for animal health and welfare, with veterinary surgeons with ongoing dairy practise work, were invited to participate in the online surveys about end‐of‐life decision‐making and euthanasia of dairy cows and calves. Separate surveys for farmers (Supporting Information S1) and veterinary surgeons (Supporting Information S2) were created on an online survey platform (www.jisc.ac.uk, Bristol, UK). The Veterinary Research Ethics Committee (VREC994) at the University of Liverpool (November 2020) approved the project before survey distribution.

Participation was encouraged by offering a chance to win one of two £50 gift cards for each cohort. Farmers were contacted about the survey through the Tesco Sustainable Dairy Group farmer forum (700 farms), social media channels such as Twitter (Agriculture and Horticulture Development Board (https://ahdb.org.uk), Animal and Plant Health Agency (https://www.gov.uk/government/organisations/animal‐and‐plant‐health‐agency) and the British Dairying electronic bulletin (6000 subscribers; of those, on average 4500 open the bulletin each week). The British Cattle Veterinary Association (1412 members) and four major corporate farm animal practices distributed the survey link to their veterinary staff in the United Kingdom.

We designed each questionnaire to last approximately 15 minutes and included questions encompassing the provision of farm standard‐operating procedures, farm staff training, veterinary intervention, culling decisions, youngstock euthanasia protocols and procedures, timeliness of decisions and subsequent events. We defined youngstock as preweaned and weaned animals up to the point of first calving. Questions required either a numerical answer, multiple‐choice answer or open‐text responses. All questions are given in the Supporting Informations S1 and S2. We aimed to reduce the risk of response bias by restricting questions to the most recent calendar year (2019), avoiding leading questions, providing interval answer options that were exhaustive. There was opportunity for respondents to elaborate on the answers provided in free‐text boxes associated with select questions and at the end of the surveys. ‘Euthanasia’ and ‘died (not euthanased)’ were the terms used in the survey in reference to animal mortality with and without euthanasia, respectively.

The initial survey included time‐to‐event questions; however, on beta‐testing of the survey by three dairy farmer clients of the University of Liverpool Farm Practice, it became apparent that few farmers are able to provide this information either because it was not recorded or very difficult to retrieve. Unfortunately, that meant that we could not address one of our original goals, which was to characterise time from disease onset to outcome: death, euthanasia or recovery.

The veterinary survey was open for approximately 19 weeks from 17 November 2020 to 31 March 2021; while the farmer survey was open for approximately 23 weeks from 16 November 2020 to 30 April 2021. At the end of the survey periods, the responses were downloaded from the survey platform into Microsoft Excel (Richmond, VA, USA) files and checked for anomalies.

### Data analysis

Statistical analyses were conducted using commercially available software (STATA 15, College Station, TX, USA). After checking for data entry errors, the data were quantitatively analysed to produce graphical and numerical summaries. Results are presented as mean ± SE unless otherwise indicated. Median averages are provided where the data are not normally distributed. Pearson pairwise correlations were performed to determine if there were any correlations among the following factors: total mortality risk within the milking herd and youngstock; the risk of death in the milking herd and youngstock; the risk of euthanasia in the milking herd and youngstock.

We calculated incidence risk (henceforth ‘risk’) as the number of events over the calendar year divided by the number of animals at risk at the start of the calendar year. We calculated the risk of youngstock euthanasia as the number of youngstock euthanased divided by the total number of youngstock owned by the farm on 1 January 2020. We performed two paired *t*‐tests to determine if there were differences between the risk of euthanasia and the risk of unassisted/natural death (death without euthanasia) among youngstock and among the milking herd, respectively. Relative risk calculations were considered inappropriate as multiple farms had zero cases of either unassisted/natural death or euthanasia. Outliers in box and whisker plots represent individual observations that were greater than 1.5 times the interquartile range above the upper quartile or below the lower quartile.

## RESULTS

**FIGURE 1:**
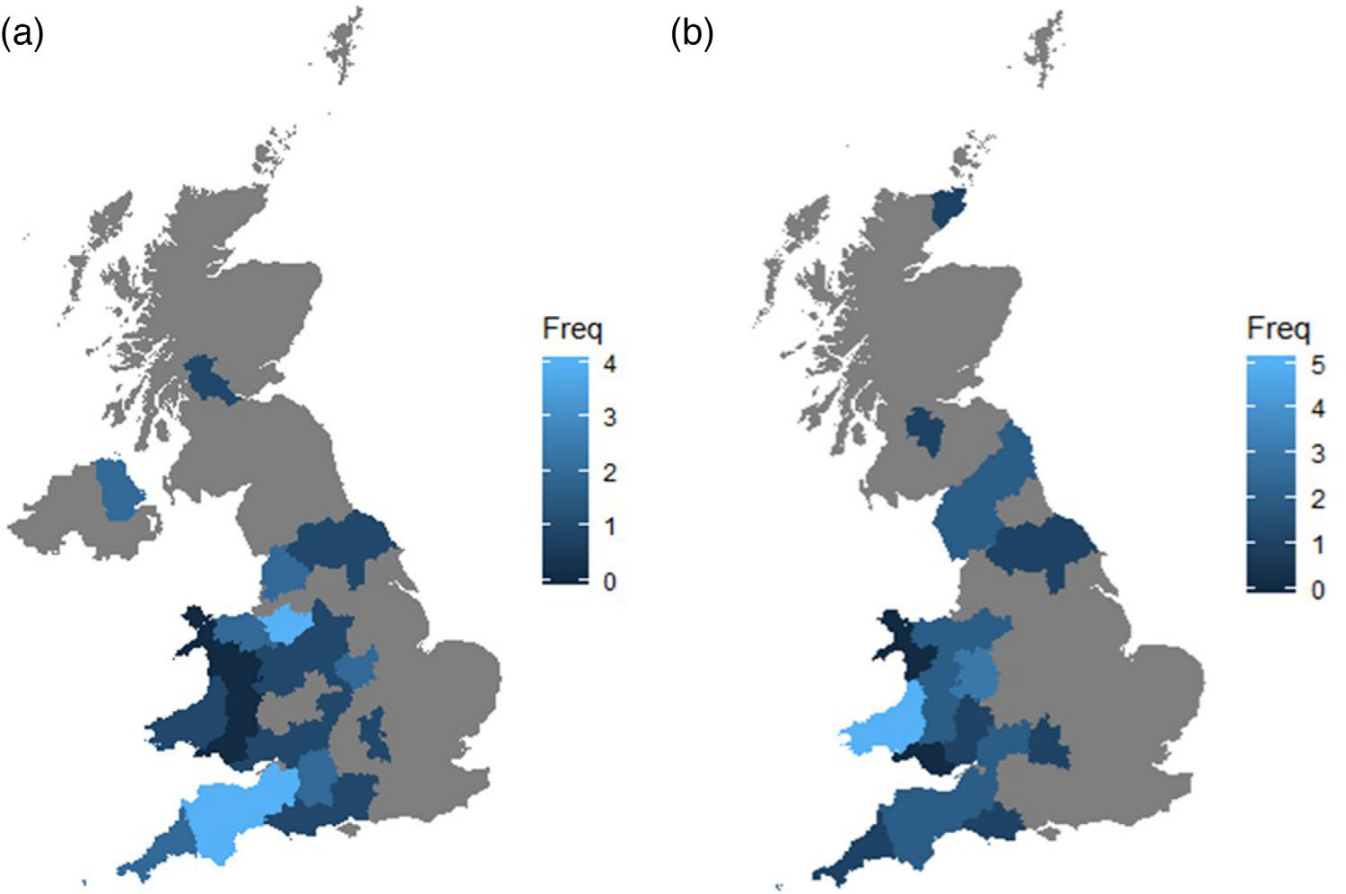
Density map showing the frequency of farms (a) and veterinary practices (b) worked at by county within the United Kingdom as reported by veterinary respondents. Grey areas represent zero.

Thirty‐seven farmers and 46 veterinarians completed the surveys in full. There were no missing data. Most respondents were located in Wales and South‐West England, with some participants located in Scotland and Northern Ireland (Figure 1).

### Survey participants

Farmer participants had a median of 220 milking animals (range 20–2000) and 139 youngstock (range 25–1000); they produced a median of 9277 L milk/cow/year (range 4227–12,600 L). The majority of farmers reported contracts with Arla or Müller (together *n* = 29, 78%). Veterinary respondents varied in their duration of dairy veterinary experience from less than 5 years to 21 or more years; the modal category of years of dairy cattle experience was 11–15 years (*n* = 14, 30%).

### Farmer responses

#### Euthanasia training

Most farms (*n* = 23 of 35, 66%) did not have anyone trained to perform euthanasia. Across all farms, only 26 of 131 farm staff (20%) had received some form of euthanasia training. The majority of farm personnel who had received training had 11 or more years of animal care experience (*n* = 21 of 26, 81%). None of the nine staff with less than 1 year of animal care experience had received training. Sixteen farms provided information about who provided euthanasia training and they could identify more than one source. A competent stockperson (*n* = 11, 69%) was the most likely source of training, followed by a veterinary surgeon (*n* = 10, 63%) and seldom a ‘book/internet/document’ (*n* = 2, 13%).

#### Euthanasia undertaking and timeliness

The majority of farmer respondents reported that euthanasia was typically performed by a fallen stock collector (*n* = 23, 62%) rather than a trained member of farm staff (*n* = 6, 22%) or a veterinary surgeon (*n* = 6, 16%). The majority of farmers reported that their farm has a standard operation procedure on the euthanasia of cattle and calves (*n* = 21, 57%).

#### Reasons for on‐farm deaths without assistance and euthanasia

Across all farms, there were 355 mortalities within the milking herd (157 unassisted/natural deaths and 198 euthanasia events) and 209 youngstock mortalities (134 unassisted/natural deaths and 75 euthanasia events). The total risk of mortality was 3.9% ± 2.5% (±SD) in the milking herd and 4.0% ± 2.8% in the youngstock. Among youngstock, the risk of unassisted/natural death (2.6% ± 0.3%) was almost double the risk of euthanasia (1.4% ± 0.3%; *p* = 0.003). The opposite, however, was true in the milking herd where the risk of euthanasia (2.3% ± 0.3%) was greater than unassisted/natural death (1.6% ± 0.2%; *p* = 0.05). There were no pairwise correlations within farms between the risk of total mortality (unassisted/natural deaths and euthanasia) in the milking herd and youngstock (*r* = 0.15), risks of unassisted/natural death in the milking herd and youngstock (*r* = 0.26) or between the risks of euthanasia in the milking herd and youngstock (*r* = 0.18).

**FIGURE 2:**
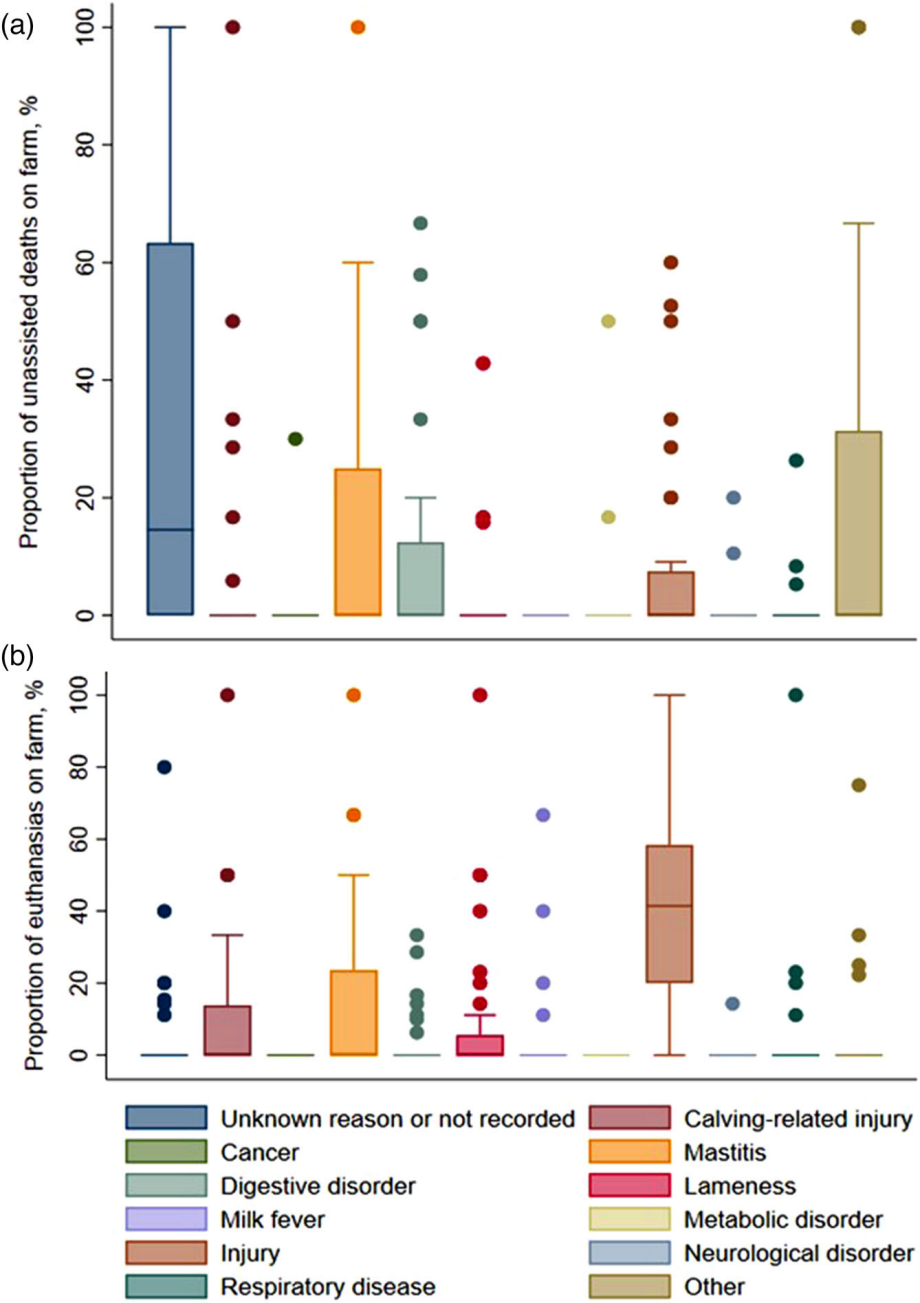
The proportion of deaths (a) and euthanasia events (b) in the milking herd (adult cows) attributable to various causes

The majority of the unassisted/natural deaths within the milking herd were attributed to ‘unknown or not recorded’ (median = 15% of milking herd deaths), with one quarter of all herds attributing between 63% and 100% of their milking herd deaths to ‘unknown or not recorded’ (Figure 2a). Less common causes of natural deaths within the milking herd included ‘other’, ‘mastitis’ and ‘digestive disorder’. ‘Other’ reasons included clostridial disease, heart attack, Johne's disease, listeria, suspected wire/ulcer and old age. Injury was by far the most common reason for euthanasia within the milking herd, with a median of 41% of all euthanasia events across farms. Mastitis, calving‐related injury and lameness were other common reasons for euthanasia in the milking herd (Figure 2b).

**FIGURE 3:**
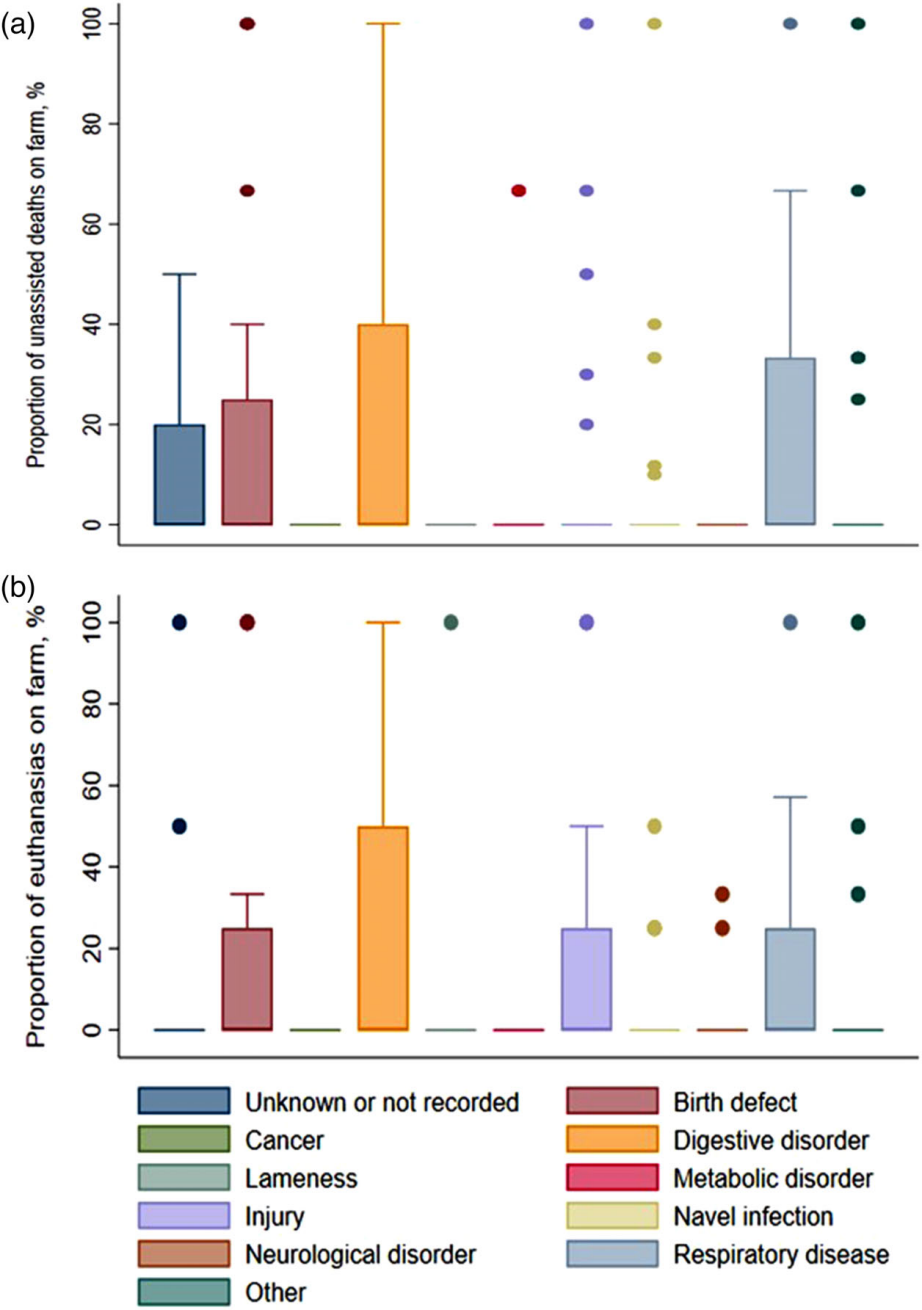
The proportion of deaths (a) and euthanasia events (b) in youngstock herd attributable to various causes

Thirty of the 37 farms had at least one youngstock death and 22 of the 37 farms euthanased at least one youngstock. Digestive and respiratory diseases were the most common causes of death among youngstock across all farms (Figure 3). Birth defects and ‘unknown or not recorded’ were also common causes of unassisted/natural deaths among youngstock. Digestive and respiratory diseases were the most common reason for youngstock euthanasia, followed by injury, birth defect, navel infection and unknown/not recorded (Figure 3).

#### Degree of difficulty experienced by farmers in making certain decisions or actions

**FIGURE 4:**
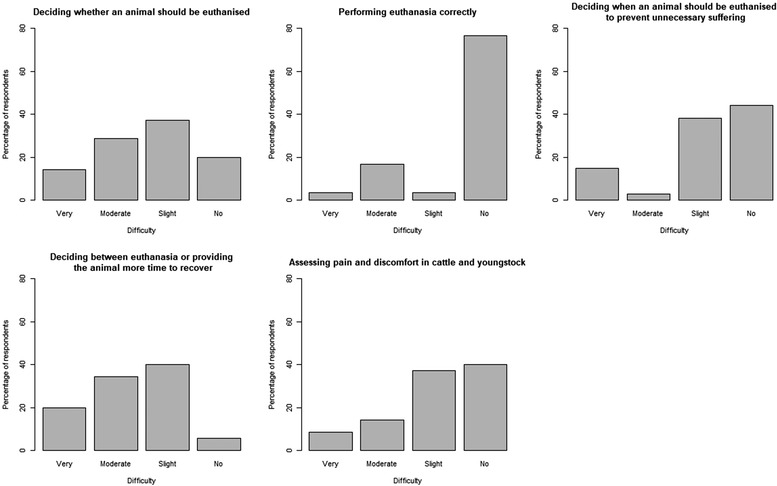
Bar charts showing the percentage of farmer respondents who found each of the five listed decisions or actions ‘very difficult’, ‘moderately difficult’, ‘slightly difficult’ or ‘not difficult’

Most farmers reported slight (37%) or moderate (29%) difficulty deciding whether an animal should be euthanased, but had no difficulty (77%) performing it correctly (Figure 4). Most farmers reported no (44%) or slight (38%) difficulty deciding when an animal should be euthanased to prevent unnecessary suffering but, paradoxically, only 6% of farmers said that they had no difficulty deciding between euthanasia or providing the animal more time to recover. Most farmers reported no (40%) or slight (37%) difficultly assessing pain and discomfort in cattle and youngstock.

#### Factors influencing farmer decisions on when an animal should be euthanased relative to the onset of disease

**FIGURE 5:**
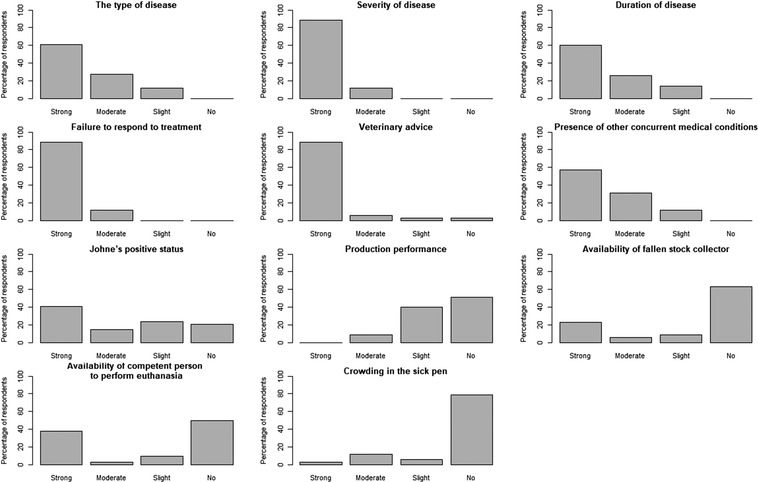
Bar charts showing factors influencing farmers’ decisions on when an animal should be euthanased relative to the onset of disease as reported by farmer respondents

‘Failure to respond to treatment’ (89%), ‘veterinary advice’ (89%) and ‘severity of disease’ (88%) were the factors most strongly influencing a farmer's decision on when an animal should be euthanased relative to the onset of disease (Figure 5). These were followed by type of disease (61%), duration of disease (60%) and presence of other concurrent medical conditions (57%). Other factors that influenced decisions on the timing of euthanasia were reported as ‘drug withdrawal’ and ‘incoming weather’. ‘Crowding in the sick pen’ (79%) had no influence on the majority of farmers and ‘production performance’ had ‘slight’ or ‘no’ influence on 91% of farmers on their decision to euthanase. The ‘availability of a fallen stock collector’ and ‘availability of competent person to perform euthanasia’ had a divergent distribution of responses, with most farmers indicating that these factors had no influence on their decision of when to euthanase (63% and 50%, respectively); however, ‘strong influence’ was the second most common answer (23% and 38%, respectively) (Figure 5).

### Veterinary surgeon responses

Forty‐six veterinary surgeons responded to the survey; most worked in England (67%) (Figure 1). Most had 11–15 years of experience with dairy cattle production (30%), followed by 1–5 years (26%), 21+ years (26%), 6–10 years (13%) and 16–20 years (4%).

The majority of veterinary surgeons did not offer euthanasia training to their clients (*n* = 34, 75%); furthermore, five (11%) did not know whether they or their veterinary practice offered euthanasia training. All the veterinary surgeons who did provide euthanasia training (*n* = 7, 15%), however, also provided guidance relating to the timing of euthanasia relative to disease onset. Free‐text responses as to how they provided this guidance included the following:

- Through discussion on how to evaluate the level of suffering the animal is under.
- At the time relative to individual case.
- With farms that have a captive bolt gun and are able to euthanase, we discuss animals on a case‐by‐case basis either in person or over the phone based on markers for non‐recovery, such as time spent recumbent or nature and degree of swelling and treatment attempted.

Almost all veterinary surgeons (96%) agreed (either ‘strongly agree’ or ‘agree’) with the following statement ‘Giving advice to farm clients regarding when to perform euthanasia is important to prevent unnecessary suffering’. The majority (92%) disagreed (either ‘strongly disagree’ or ‘disagree’) with the following statement ‘It is solely up to the farm client to make the decision around the timing of euthanasia’.

#### Veterinary surgeons’ confidence in their farmer clients’ timeliness to euthanasia

**FIGURE 6:**
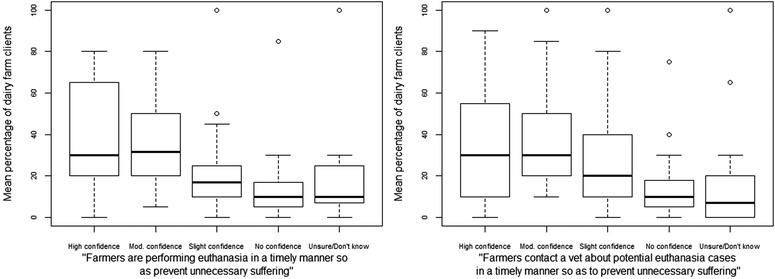
Box and whisker plots showing veterinary surgeons’ confidence in the following statements: ‘Farmers are performing euthanasia in a timely manner so as to prevent unnecessary suffering’ (left) and ‘Farmers contact a vet about potential euthanasia cases in a timely manner so as to prevent unnecessary suffering’ (right)

In general, veterinary surgeons were more confident than not, that ‘Farmers are performing euthanasia in a timely manner so as to prevent unnecessary suffering’. On average (median), veterinary surgeons had ‘high confidence’ (30% of farms) and ‘moderate confidence’ (32% of farms) that their farm clients were performing euthanasia in a timely manner so as to prevent unnecessary suffering (Figure 6). Veterinary surgeons had ‘no confidence’ that timely euthanasia was being performed in 10% of their herds and were ‘unsure’ about another 10% of their herds.

Veterinary surgeons’ responses to the following statement followed a similar pattern ‘Farmers contact a vet about potential euthanasia cases in a timely manner so as to prevent unnecessary suffering’. Veterinary surgeons had ‘high confidence’ (30% of farms) or ‘moderate confidence’ (30% of farms) in the majority of their clients, but they had ‘no confidence’ that they were being contacted in a timely manner in 10% of their herds and were ‘unsure’ about another 7% of their herds.

#### Timeframe to euthanasia of a first‐lactation heifer currently in milk (that is otherwise healthy) relative to the onset of disease

Veterinary surgeons agreed on the timeframe of euthanasia for some conditions (Table 1). For example, 89% agreed on ‘within 6 hours’ for ‘intractable uterine prolapse’. Other conditions saw no consensus in the timeframe of euthanasia. For example, a first‐lactation heifer with ‘toxic mastitis’ where around a fifth to a quarter of respondents reported each of ‘within 24 hours’, ‘within 2 days’, ‘within 3–5 days’ and ‘would not recommend’ (Table 1).

**TABLE 1:** When veterinary surgeons believed a first‐lactation heifer currently in milk (that is otherwise healthy) should be euthanised relative to the onset of each disease/condition

	**Time to euthanasia recommendation, % of dairy veterinarians**					
**Presenting information**	**<6 hours**	**<24 hours**	**<2 days**	**3–5 days**	**6+ days**	**Would not recommend**
Down cow with suspected calving injury (not responded to milk fever treatment)	2	7	15	52	24	0
Severe lameness (mobility score 3)	0	7	7	13	57	17
Chronic diarrhoea	0	7	7	15	50	22
Toxic mastitis	2	24	26	26	0	22
Intractable vaginal prolapse	13	39	15	7	7	17
Intractable uterine prolapse	89	9	0	0	0	2
Down cow (hip dislocation)	72	22	4	2	0	0
Oedematous brisket region, cough, jugular vein distension and pulsation	17	39	20	17	2	4

#### Timeframe to euthanasia of a dairy calf (that is otherwise healthy) relative to the onset of disease

Again, veterinary surgeons agreed on the timeframe to euthanasia for some conditions (Table 2). For example, 65% agreed on ‘within 6 hours’ for a calf that is ‘depressed and non‐ambulatory with tachycardia, tachypnoea, hyperaemia of mucous membranes and scleral injection’. Other conditions saw no consensus in the timeframe of euthanasia. For example, ‘suspected ventricular septal defect’ where around a fifth to a quarter of respondents reported each of ‘within 6 hours’, ‘within 24 hours’, ‘within 2 days’ and ‘6+ days’ (Table 2). Duration of veterinary experience did not influence time‐to‐euthanasia decisions for either scenario. Free‐text comments from veterinary surgeons relating to the ‘timeliness of euthanasia of dairy cattle and youngstock’ largely centred on response to treatment, the level of on‐farm care (more likely to euthanase sooner if regular lifting of non‐ambulatory cows or provision of analgesia was questionable, for example), prognosis and the economics of sustained treatment.

**TABLE 2:** When veterinary surgeons believed a dairy calf that was otherwise healthy should be euthanased relative to the onset of each disease/condition

	**Time to euthanasia recommendation, % of dairy veterinarians**					
**Presenting information**	**<6 hours**	**<24 hours**	**<2 days**	**3–5** **days**	**6+ days**	**Would not recommend**
Joint illness	0	4	9	22	30	35
Chronic pneumonia	7	15	4	11	47	15
Chronic bloat	2	15	13	17	17	35
Cleft palate	43	37	2	7	4	7
Chronic diarrhoea	7	7	9	20	35	23
Suspected ventricular septal defect	20	28	20	4	22	7
Depressed and non‐ambulatory with tachycardia, tachypnoea, hyperaemia of mucous membranes and scleral injection	65	24	9	0	0	2

## DISCUSSION

The results of this survey highlight some of the challenges faced by farmers and veterinary surgeons when making end‐of life decisions on dairy farms. As shown by the results, the issues influencing euthanasia decisions are multifactorial and sometimes complex. In general, farmers were confident that euthanasia was performed correctly and were confident in assessing pain and distress in their animals. Deciding whether an animal should be euthanased or provided more time to recover appeared to be more difficult. Even veterinary surgeons failed to concur on when animals should be euthanased in more complex diseases, such as toxic mastitis, but agreed on more clear‐cut scenarios, such as intractable uterine prolapse.

The results indicated that the majority of British dairy farms were dependent on external personnel, typically fallen stock collectors, for euthanising animals on the farm because they did not have anyone ‘in‐house’ trained to perform euthanasia. Just 22% of British farms in our study performed their own euthanasia compared to 92% of US dairy farms^4^ and 45% of Danish farmers.^5^ Interestingly, the same study of Danish farmers found that 58% of cow mortalities were euthanased,^5^ while another study of Danish dairy farms found that only 17% of cows that died were euthanased.^6^ Just 40% of cow deaths on US dairy farms were attributed to euthanasia in a NAHMS study^4^ compared to 56% of cows in our survey. These findings suggest that having the on‐farm capability to euthanase does not necessarily translate to fewer animals dying without the benefit of euthanasia. While the reasons for this are likely multifactorial, the emotional strain and sense of failure associated with killing your own animals^7^ is likely a major component that may lead to fewer animals being euthanased, than if someone external to the farm were to routinely perform the euthanasia.

While there are advantages of having the expertise of a licensed slaughterman to perform euthanasia of livestock, there are also clear drawbacks such as the necessity to wait for the fallen stock collector to arrive and the ability of the fallen stock collector to arrive due to inclement weather for example. The Red Tractor dairy farm assurance standards (Version 5.0; www.redtractorassurance.org.uk/standards/animal‐health‐and‐welfare‐10/) states that a ‘competent person is available to production sites as soon as possible (normally within 60‐minute drive) in order to deal with emergency cases promptly and prevent unnecessary suffering’. While this is an admirable guideline, it is unclear how closely farmers are able to conform to it. The ‘availability of a fallen stock collector’ and ‘availability of competent person to perform euthanasia’ had a ‘strong influence’ on 23% and 38% of farmers, respectively, on when an animal should be euthanased relative to the onset of disease. Farm geographical remoteness from the nearest fallen stock collector is probably a key factor influencing the ability of farms to perform euthanasia if they do not have trained staff.

The proportion of farms (57%) that reported having a standard operation procedure for the euthanasia of cattle and calves was lower than expected. The Red Tractor farm assurance scheme, of which over 95% of British dairy farms are reportedly members, requires that a euthanasia policy is included in the health plan. This suggests that either 43% of farmer participants were not representative of the dairy industry, failed to comply with their health plan, or may have a farm euthanasia policy, but those who answered the survey were not familiar with it. Some veterinary surgeons, however, have called into question the utility of relying on written euthanasia protocols and believe that they should be accompanied by interactive on‐farm training to improve engagement with the protocol and improve comprehension of a protocol that is typically written using veterinary terminology.^7^ Some have suggested that the combination of training and protocols are an integral part of the euthanasia decision‐making process, largely because it provides an opportunity to address the emotional stress and bereavement that are not a component of the written protocol.^8^

The average mortality risk of 4% in the milking herd and 4% in youngstock in our study was slightly lower than reports for the US,^4^ Canadian^9^ and Danish^5^ dairy farms, while similar to a survey of Italian dairy farms (3.7%).^10^ In our study, similar to US dairy herds,^4^ youngstock were less likely to be euthanased than animals in the milking herd. The reasons for this are likely multifactorial: failure of passive transfer and an immature immune system may predispose youngstock to diseases with a more rapid onset and greater severity than cows; youngstock are also typically less visible than cows in the milking herd as they are housed at the periphery of the farm or grazing distant pastures.

The varied responses of dairy cattle veterinary surgeons regarding when euthanasia should be performed relative to disease onset are largely unsurprising given that the decisions are typically multifactorial. In general, however, there was a tendency to try to treat even if the prognosis was poor. For example, 52% of veterinary surgeons elected not to euthanase a non‐ambulatory cow with a suspected calving injury until 3–5 days later; while 24% elected to wait for 6 or more days. The evidence, however, indicates that only 8% of cows that are non‐ambulatory for at least 24 hours recover.^11^ An evidence‐based decision support tool could help guide veterinary surgeons make timely euthanasia decisions in instances where an inherent bias toward treatment may cloud rationale decision‐making.

An original goal of this project was to characterise time from disease onset to outcome: death, euthanasia or recovery. It quickly became apparent, however, that these data, along with whether animals were non‐ambulatory before death or euthanasia, were largely unavailable or were difficult to retrieve from farm records. This presents an important animal welfare information void that hinders the development of an evidence‐based end‐of‐life decision‐support framework for use on dairy farms. The latter would require a large, multi‐farm cohort study in which farmers record animal health events and treatment information in more detail than is currently required by law.

Failure to determine or record the cause of death was another information void identified in our study. The most cited category (15%) for unassisted/natural deaths within the milking herd were ‘unknown reasons’. A similar proportion of cow deaths due to unknown causes has been reported for US (12%),^4^ Danish (15%)^6^ and Italian (15%)^10^ dairy herds. Furthermore, deaths attributed to specific causes may be incorrect as farmer perceptions alone, rather than a definitive clinical diagnosis, can lead to cause of death misclassifications.^12^, ^13^ Postmortem examinations are sometimes not warranted where the cause of death is clear, although valuable herd management information could be gained in many situations such as confirmation of a tentative clinical diagnosis that may require collection of samples for pathological testing, where the death was sudden, unusual or no antemortem signs were observed.^14^ Like all surveys that require self‐reporting of retrospective events, we must also acknowledge the potential for recall bias in our results.

Unfortunately, the number of responses was lower than anticipated; although the farmer participants that did respond had a similar number of livestock and milk yields to median values reported for British herds,^15^ so we believe the results to be representative of the British dairy industry. The low participation rate may have been attributable to the survey being distributed during COVID‐19 national lockdowns, which limited our ability to distribute the survey through national media and ‘survey fatigue’ as many researchers switched to non‐contact‐based research methods. Alternatively, farmers and veterinary surgeons may have felt discouraged to participate as they lack or were unable to retrieve sufficiently detailed records of dairy cattle deaths and euthanasia events to feel that their participation was beneficial.

The results of this study highlighted key aspects of end‐of‐life decision‐making that dairy farmers and veterinary surgeons are accomplishing successfully or need improving. We believe that an evidence‐based decision‐support framework would empower farmers and veterinary surgeons with the ability to make better‐informed and timelier decisions. This would improve the quality of life of dairy cattle and youngstock by preventing unnecessary suffering. Creation of this framework, however, requires the collection and analysis of antemortem details, cause‐of‐death and time‐to‐event data, which are not routinely collected on farms or are difficult to retrieve.

## AUTHOR CONTRIBUTIONS

Joseph Neary and Robert Smith designed the study. Joseph Neary, Cherry Bedford and Robert Smith wrote the surveys. Joseph Neary and Cherry Bedford analysed the data and generated the figures. Joseph Neary and Robert Smith wrote the manuscript.

## CONFLICTS OF INTEREST

The authors declare they have no conflicts of interest.

## FUNDING INFORMATION

This study was funded by the Animal Welfare Foundation (AWF). AWF is a fundraising and grant giving charity (charity number 1192203) directed by veterinary and animal welfare professionals, which uses scientific knowledge to improve the welfare of animals through research, education and debate. More information can be found at www.animalwelfarefoundation.org.uk

## ETHICS STATEMENT

The authors confirm that the ethical policies of the journal, as noted on the journal's author guidelines page, have been adhered to. The Veterinary Research Ethics Committee (VREC994) at the University of Liverpool (November 2020) approved the project before survey distribution.

## Supporting information

Supporting Information S1 Farm survey

Supporting Information S2 Vet survey

## Data Availability

Data are available on request from the authors.

## References

[1] Rioja‐Lang FC , Connor M , Bacon HJ , Lawrence AB , Dwyer CM . Prioritization of farm animal welfare issues using expert consensus. Front Vet Sci. 2020;6:495. doi:10.3389/fvets.2019.00495 31998770 PMC6967597

[2] Walker JB , Roman‐Muniz IN , Edwards‐Callaway LN . Timely euthanasia in the United States dairy industry–challenges and a path forward. Animals. 2019;10(1):71. doi:10.3390/ani10010071 31906056 PMC7022783

[3] Opinion on the welfare of animals killed on‐farm. Farm Animal Welfare Committee . Accessed October 14, 2022. https://assets.publishing.service.gov.uk/government/uploads/system/uploads/attachment_data/file/695225/fawc‐opinion‐welfare‐of‐animals‐killed‐on‐farm‐march2018.pdf

[4] Dairy 2014 Health and Management Practices on U.S. Dairy Operations, 2014. USDA . Accessed October 14, 2022. https://www.aphis.usda.gov/animal_health/nahms/dairy/downloads/dairy14/Dairy14_dr_PartIII.pdf

[5] Thomsen PT , Kjeldsen AM , Sørensen JT , Houe H . Mortality (including euthanasia) among Danish dairy cows (1990–2001). Prev Vet Med. 2004;62(1):19–33. doi:10.1016/j.prevetmed.2003.09.002 15154682

[6] Thomsen PT , Sørensen JT . Factors affecting the risk of euthanasia for cows in Danish dairy herds. Vet Rec. 2009;165(2):43–5. doi:10.1136/vetrec.165.2.43 19596674

[7] Wagner BK , Cramer MC , Fowler HN , Varnell HL , Dietsch AM , Proudfoot KL et al. Determination of dairy cattle euthanasia criteria and analysis of barriers to humane euthanasia in the United States: the veterinarian perspective. Animals. 2020;10(6):1051. doi:10.3390/ani10061051 32570866 PMC7341486

[8] Edwards‐Callaway L , Simpson H , Román‐Muñiz N , Cramer C , Mijares S , Stallones L , et al. Preliminary exploration of weekly peer group discussions as a strategy for coping with feelings associated with euthanasia in dairy caretakers. Int J Environ Res Public Health. 2022;19(4):2177. doi:10.3390/ijerph19042177 35206363 PMC8872095

[9] Roche SM , Genore R , Renaud DL , Shock DA , Bauman C , Croyle S , et al. Short communication: describing mortality and euthanasia practices on Canadian dairy farms. J Dairy Sci. 2020;103(4):3599–605. doi:10.3168/jds.2019-17595 32089307

[10] Fusi F , Angelucci A , Lorenzi V , Bolzoni L , Bertocchi L . Assessing circumstances and causes of dairy cow death in Italian dairy farms through a veterinary practice survey (2013–2014). Prev Vet Med. 2017;137:105–8. doi:10.1016/j.prevetmed.2017.01.004 28089290

[11] Green AL , Lombard JE , Garber LP , Wagner BA , Hill GW . Factors associated with occurrence and recovery of nonambulatory dairy cows in the United States. J Dairy Sci. 2008;91(6):2275–83. doi:10.3168/jds.2007-0869 18487650

[12] McConnel CS , Garry FB , Lombard JE , Kidd JA , Hill AE , Gould DH . A necropsy‐based descriptive study of dairy cow deaths on a Colorado dairy. J Dairy Sci. 2009;92(5):1954–62. doi:10.3168/JDS.2008-1505 19389952

[13] Küker S , Faverjon C , Furrer L , Berezowski J , Posthaus H , Rinaldi F et al. The value of necropsy reports for animal health surveillance. BMC Vet Res. 2018;14(1):191. doi:10.1186/s12917-018-1505-1 29914502 PMC6006731

[14] Mason GL , Madden DJ . Performing the field necropsy examination. Vet Clin North Am Food Anim Pract. 2007;23(3):503–26. doi:10.1016/j.cvfa.2007.07.006 17920459

[15] Hanks J , Kossaibati M . Key performance indicators for the national UK dairy herd. A study of herd performance in 500 Holstein/Friesian herds for the year ending 31st August 2020. University of Reading; November 2020. Accessed November 2, 2022. https://panlivestock.com/wp‐content/uploads/2022/05/NMR500Herds‐Aug2020.pdf

