# Supplementary material for: End‐of‐life decision‐making of dairy cattle and calves: A survey of British farmers and veterinary surgeons
Source: Vet Rec Open. 2022 Nov 25;9(1):e51. doi: 10.1002/vro2.51 (PMC9695751; doi:10.1002/vro2.51)
Supplement: Supplementary file 1 — Supporting Information S1 Farm survey [file VRO2-9-e51-s002.pdf]

Euthanasia survey farmers

Page 1: Introduction

You are being invited to participate in a research study. Before you decide whether to participate, it is important for you to understand why the research is being done and what it will involve. Please take time to read the following information carefully and feel free to ask us if you would like more information or if there is anything that you do not understand.

Dairy farmers with responsibility for animal health and welfare are invited to participate in the following survey about end-of-life decision making and euthanasia of dairy cows and calves. The survey is funded by the Animal Welfare Foundation. The purpose of the study is to better understand how farmers determine when an animal should be euthanised and the factors that influence their decision.

The survey consists of 17 short questions and takes approximately 15 minutes to complete. Please provide a phone number at the end of the survey if you wish to be entered into the draw to win one of two £50 gift cards. The telephone numbers will only be used to contact the prize draw winners and will be stored separately to your survey answers. This ensures that your survey responses are anonymous and confidential.

Data are to be held on a secure, central University of Liverpool computer system until completion of the project in accordance with UK General Data Protection Regulations. The results of the survey will be analysed as part of a research project and published in a scientific journal and other resources for knowledge transfer. By agreeing to take part in this survey, you consent to your data being used in this way. If you have any questions, concerns, or feedback about this survey please contact Dr Joseph Neary at j.neary@liverpool.ac.uk

The University processes personal data as part of its research and teaching activities in accordance with the lawful basis of 'public task', and in accordance with the University's purpose of "advancing education, learning and research for the public benefit.

Under UK data protection legislation, the University acts as the Data Controller for personal data collected as part of the University's research. The Principal Investigator acts as the Data Processor for this study, and any queries relating to the handling of your personal data can be sent to Dr. Neary.

Further information on how your data will be used can be found in the table below.

How will my data be collected?	By participating in the online survey
How will my data be stored?	Storage of data on a secure network drive of the University of Liverpool
How long will my data be stored for?	Raw data stored until completion of project and potential publication, expected February 2022.
What measures are in place to protect the security and confidentiality of my data?	Data will be anonymised to remove information that readily identifies you. Storage is password protected.

Will my data be anonymised?	Yes. Contact information is separated from responses.
How will my data be used?	Analysis in statistical and database software.
Who will have access to my data?	Supervisors (Joseph Neary and Rob Smith) and research assistant (Cherry Bedford)
Will my data be archived for use in other research projects in the future?	Any further storage and use of data after February 2022 will be subject to further ethical review.
How will my data be destroyed?	Deleted from the server.

Page 2: Questions about the farm

Size of farm on 1st January 2020 (estimated):

Milking herd and dry cows (cows and heifers)

Please enter a whole number (integer).

All youngstock (preweaned and weaned)

Page 3: Questions about the farm

Country where farm is located

County where farm is located

County where farm is located

County where farm is located

County where farm is located

Who is your milk buyer?

If you selected Other, please specify:

Do you have a supermarket aligned contract?

If you selected Other, please specify:

What was the rolling herd average for milk production in 2020 (in L/year)

Please enter a number.

Page 4: Farm staff/ standard operating procedures

Farm staff

Please indicate the number of farm staff involved in animal care according to the following years of animal care experience AND whether they have or have not received training in the euthanasia of cattle and calves:

More info

	Number of staff that HAVE received euthanasia training	Number of staff that HAVE NOT received euthanasia training
Less than one year animal care experience	0	0
One to five years animal care experience	0	0
Six to ten years animal care experience	0	0
More than ten years animal care experience	0	0

Who has provided training in the euthanasia of dairy cattle and calves? (Check all that apply)

- ☐ Veterinarian
- ☐ A competent stockperson
- ☐ External course
- ☐ Book/internet/document
- ☐ Other

If you selected Other, please specify:

Who was the course organiser?

Can you provide more details?

Euthanasia of dairy cattle and calves is typically performed by:

If you selected Other, please specify:

Standard operating procedure

Does the farm have a standard operating policy on the euthanasia of cattle and calves?

- Yes
- No
- Don't know

Does the standard operating procedure include a timeframe from the onset of disease for when euthanasia should occur?

- Yes
- No

- Don't know
- Other

If you selected Other, please specify:

Page 5: Animals that died on the farm in 2020

How many animals in the milking herd and dry cows **died** (were not euthanised) on the farm in 2020 due to the following reasons:

	Number of deaths (not euthanised)
Unknown reason or not recorded	0
Calving-related injury	0
Cancer	0
Mastitis	0
Digestive disorder (diarrhoea, DA, etc)	0
Lameness	0
Milk fever	0
Metabolic disorder (ketosis)	0
Injury (inc. broken leg)	0
Neurological disorder	0
Respiratory disease	0
Other	0

If you have another reason/s - please specify

How many of the following **youngstock** animals **died** (not euthanised) on the farm in 2020 due to the following reasons

	Number of deaths (not euthanised)
Unknown reason or not recorded	0

Birth defect	0
Cancer	0
Digestive disorder (diarrhoea, DA, etc)	0
Lameness	0
Metabolic disorder (ketosis)	0
Injury (inc. broken leg)	0
Navel infection	0
Neurological disorder	0
Respiratory disease	0
Other	0

If you have another reason/s - please specify

Page 6: Animals that were euthanised on the farm in 2020

How many animals in the milking herd and dry cows were **euthanised** on the farm in 2020 due to the following reasons:

	Number euthanised
Unknown reason or not recorded	0
Calving-related injury	0
Cancer	0
Mastitis	0
Digestive disorder (diarrhoea, DA, etc)	0
Lameness	0
Milk fever	0
Metabolic disorder (ketosis)	0
Injury (inc. broken leg)	0
Neurological disorder	0
Respiratory disease	0
Other	0

If you have another reason/s - please specify

How many of the following **youngstock** animals were **euthanised** on the farm in 2020 due to the following reasons

	Number euthanised
Unknown reason or not recorded	0

Birth defect	0
Cancer	0
Digestive disorder (diarrhoea, DA, etc)	0
Lameness	0
Metabolic disorder (ketosis)	0
Injury (inc. broken leg)	0
Navel infection	0
Neurological disorder	0
Respiratory disease	0
Other	0

If you have another reason/s - please specify

Page 7: Decision making

Indicate the level of difficulty experienced in making the following decisions or actions as they relate to your farm

Please don't select more than 1 answer(s) per row.

	Very difficult	Moderate difficulty	Slight difficulty	No difficulty
Deciding whether an animal should be euthanised	☐	☐	☐	☐
Performing euthanasia correctly	☐	☐	☐	☐
Deciding when an animal should be euthanised to prevent unnecessary suffering	☐	☐	☐	☐
Deciding between euthanasia or providing the animal more time to recover	☐	☐	☐	☐
Assessing pain and discomfort in cattle and youngstock	☐	☐	☐	☐

Indicate to what extent the following factors influence your decision on **when** an animal should be euthanised relative to the onset of disease:

Please don't select more than 1 answer(s) per row.

	Strong influence	Moderate influence	Slight influence	No influence
The type of disease	☐	☐	☐	☐
Severity of disease	☐	☐	☐	☐
Duration of disease	☐	☐	☐	☐
Failure to respond to treatment	☐	☐	☐	☐
Veterinary advice	☐	☐	☐	☐
Presence of other concurrent medical conditions such as mastitis, emaciation etc.	☐	☐	☐	☐

Johne’s positive status	☐	☐	☐	☐
Production performance	☐	☐	☐	☐
Availability of fallen stock collector	☐	☐	☐	☐
Availability of competent person to perform euthanasia	☐	☐	☐	☐
Crowding in the sick pen	☐	☐	☐	☐
Other	☐	☐	☐	☐

If you have another reason/s - please specify

Page 8: Opinions

Do you feel there is a conflict between animal welfare and commercial interest?

- Yes
- No

Please add any comments here

If you have any comments about the timeliness of euthanasia of cattle and youngstock or comments on any of your answers above, please add them here

Would you like to be entered into a draw for a chance to win one of two £50 gift cards

- Yes
- No

Telephone number

Please enter a valid phone number.

Page 9: Final page

Thank you for taking part in the survey. If you have any questions, concerns, or feedback about this survey please contact Dr Joseph Neary at j.neary@liverpool.ac.uk

---

Key for selection options

3 - Country where farm is located

England
Northern Ireland
Scotland
Wales

4 - Who is your milk buyer?

Arla
County Milk Products
Dairy Crest
Dale Farm
First Milk
Glandbia Cheese
Meadow Foods
Müller
Ornua
Yeo Valley
Other

5 - Do you have a supermarket aligned contract?

No aligned contract
Tesco Sustainable Dairy Group
Sainsbury's Dairy Development Group
Waitrose and Partners
Marks and Spencer
The Co-operative
Other

8 - Euthanasia of dairy cattle and calves is typically performed by:

A farmer/competent stock person – only one person on the farm performs euthanasia
A farmer/competent stock person – two or more people on the farm perform euthanasia

Veterinarian
Fallen stock collector
Other
