# Supplementary material for: End‐of‐life decision‐making of dairy cattle and calves: A survey of British farmers and veterinary surgeons
Source: Vet Rec Open. 2022 Nov 25;9(1):e51. doi: 10.1002/vro2.51 (PMC9695751; doi:10.1002/vro2.51)
Supplement: Supplementary file 2 — Supporting Information S2 Vet survey [file VRO2-9-e51-s001.pdf]

# Euthanasia survey Vets

## Page 1: Introduction

*You are being invited to participate in a research study. Before you decide whether to participate, it is important for you to understand why the research is being done and what it will involve. Please take time to read the following information carefully and feel free to ask us if you would like more information or if there is anything that you do not understand.*

Veterinarians with ongoing dairy practise work are invited to participate in the following survey about end-of-life decision making and euthanasia of dairy cows and calves. The survey is funded by the Animal Welfare Foundation. The purpose of the study is to better understand how veterinarians determine when an animal should be euthanised and the factors that influence their decision.

The survey consists of eight questions and takes approximately 10-15 minutes to complete. Please provide a phone number at the end of the survey if you wish to be entered into the draw to win one of two £50 gift cards. The telephone numbers will only be used to contact the prize draw winners and will be stored separately to your survey answers. This ensures that your survey responses are anonymous and confidential.

Data are to be held on a secure, central University of Liverpool computer system until completion of the project in accordance with UK General Data Protection Regulations. The results of the survey will be analysed as part of a research project and published in a scientific journal and other resources for knowledge transfer. By agreeing to take part in this survey, you consent to your data being used in this way. If you have any questions, concerns, or feedback about this survey please contact Dr Joseph Neary at j.neary@liverpool.ac.uk

*The University processes personal data as part of its research and teaching activities in accordance with the lawful basis of 'public task', and in accordance with the University's purpose of "advancing education, learning and research for the public benefit.*

*Under UK data protection legislation, the University acts as the Data Controller for personal data collected as part of the University's research. The Principal Investigator acts as the Data Processor for this study, and any queries relating to the handling of your personal data can be sent to Dr. Neary.*

*Further information on how your data will be used can be found in the table below.*

| | |
|---|---|
| How will my data be collected? | By participating in the online survey |
| How will my data be stored? | Storage of data on a secure network drive of the University of Liverpool |
| How long will my data be stored for? | Raw data stored until completion of project and potential publication, expected February 2022. |
| What measures are in place to protect the security and confidentiality of my data? | Data will be anonymised to remove information that readily identifies you. Storage is password protected. |
| Will my data be anonymised? | Yes. Contact information is separated from responses. |

| | |
|---|---|
| How will my data be used? | Analysis in statistical and database software. |
| Who will have access to my data? | Supervisors (Joseph Neary and Rob Smith) and research assistant (Cherry Bedford) |
| Will my data be archived for use in other research projects in the future? | Any further storage and use of data after February 2022 will be subject to further ethical review. |
| How will my data be destroyed? | Deleted from the server. |

# Page 2: Questions about the veterinary practice

Country where veterinary practice is located

County where veterinary practice is located

County where veterinary practice is located

County where veterinary practice is located

County where veterinary practice is located

How many years of veterinary experience with dairy cattle production do you have?

- 1 – 5 years
- 6 – 10 years
- 11 – 15 years
- 16 – 20 years
- 21+ years

# Page 3: Questions about euthanasia training

Do you offer dairy cattle and youngstock euthanasia training to your dairy farm clients?

Do you provide guidance on when is the appropriate time relative to disease onset to perform euthanasia?

Please describe how you provide guidance

Please explain why you do not provide guidance

Indicate your level of agreement with the following statement:

Please don't select more than 1 answer(s) per row.

| | Strongly agree | Agree | Disagree | Strongly disagree |
|---|---|---|---|---|
| Giving advice to farm clients regarding when to perform euthanasia is important to prevent unnecessary suffering | ☐ | ☐ | ☐ | ☐ |

| | | | | |
|---|---|---|---|---|
| It is solely up to the farm client to make the decision around the timing of euthanasia | ☐ | ☐ | ☐ | ☐ |

# Page 4: Questions about your dairy farm clients

Please fill in the following table with the percentage of your dairy farm clients (each row should total 100%):

| | High confidence (%) | Moderate confidence (%) | Slight confidence (%) | No confidence (%) | Unsure/ Don't know |
|---|---|---|---|---|---|
| Farmers are performing euthanasia in a timely manner so as prevent unnecessary suffering | | | | | |
| Farmers contact a vet about potential euthanasia cases in a timely manner so as to prevent unnecessary suffering | | | | | |

## Page 5: Questions about the timing of euthanasia

Please indicate a timeframe for when you would likely perform or recommend euthanasia of a 1st lactation heifer currently in milk (that is otherwise healthy) relative to the onset of disease for the following conditions:

| | Within 6 hours | Within 24 hours | Within 2 days | Within 3 - 5 days | 6+ days | Would not recommend |
|---|---|---|---|---|---|---|
| Down cow with suspected calving injury (not responded to milk fever treatment) | ○ | ○ | ○ | ○ | ○ | ○ |
| Severe lameness (mobility score 3) | ○ | ○ | ○ | ○ | ○ | ○ |
| Chronic diarrhoea | ○ | ○ | ○ | ○ | ○ | ○ |
| Toxic mastitis | ○ | ○ | ○ | ○ | ○ | ○ |
| Intractable vaginal prolapse | ○ | ○ | ○ | ○ | ○ | ○ |
| Intractable uterine prolapse | ○ | ○ | ○ | ○ | ○ | ○ |
| Down cow (hip dislocation) | ○ | ○ | ○ | ○ | ○ | ○ |
| Oedematous brisket region, cough, jugular vein distension and pulsation. | ○ | ○ | ○ | ○ | ○ | ○ |

Please indicate a timeframe for when you would likely perform or recommend euthanasia of a dairy calf that is otherwise healthy relative to the onset of disease for the following conditions:

| | Within 6 hours | Within 24 hours | Within 2 days | Within 3 - 5 days | 6+ days | Would not recommend |
|---|---|---|---|---|---|---|
| Joint ill | ○ | ○ | ○ | ○ | ○ | ○ |
| Chronic pneumonia | ○ | ○ | ○ | ○ | ○ | ○ |
| Chronic bloat | ○ | ○ | ○ | ○ | ○ | ○ |
| Cleft palate | ○ | ○ | ○ | ○ | ○ | ○ |
| Chronic diarrhoea | ○ | ○ | ○ | ○ | ○ | ○ |
| Suspected ventricular septal defect | ○ | ○ | ○ | ○ | ○ | ○ |

| | | | | | | |
|---|---|---|---|---|---|---|
| Depressed and nonambulatory with tachycardia, tachypnea, hyperemia of mucous membranes and scleral injection. | ○ | ○ | ○ | ○ | ○ | ○ |

# Page 6: Comments

If you have any comments about the timeliness of euthanasia of dairy cattle and youngstock or comments on any of your answers above, please add them here:

To be entered into the prize draw to win a £50 gift card, please provide a telephone number below. This will only be used to contact the winner.

Please enter a valid phone number.

# Page 7: Final page

Thank you for taking part in the survey. If you have any questions, concerns, or feedback about this survey please contact Dr Joseph Neary at j.neary@liverpool.ac.uk

## Key for selection options

**1 - Country where veterinary practice is located**

England
Northern Ireland
Scotland
Wales

**3 - Do you offer dairy cattle and youngstock euthanasia training to your dairy farm clients?**

Yes
No
Don't know
